# Where do our students go? A blueprint for quantifying the local retention effect and its reach by tracking the career paths of medical students

**DOI:** 10.3205/zma001855

**Published:** 2026-06-15

**Authors:** Robin Herbrechter, Stefanie Mattern, Sophie-Charlotte Rosenberger, Annika Haupt, Marzellus Hofmann

**Affiliations:** 1 Witten/Herdecke University (UW/H), Department of Human Medicine, Witten, Germany

**Keywords:** medical education, medical faculty, career choice, ambulatory care, stay rate, pipeline effect, postgraduate follow-up, physician density, GIS, alumni density

## Abstract

**Objectives:**

Demographic and structural changes in the healthcare sector have led to an increasing shortage of physicians in Germany in recent years, especially in rural areas. Medical schools are increasingly making curricular adjustments to enhance the appeal of urgently needed positions in the healthcare sector. Graduate tracking is essential for evaluating curricula and optimization efforts, and therefore for the targeted improvement of healthcare provision.

**Methods:**

Data on the professional activities of 333 medical graduates of the University of Witten/Herdecke (UW/H) were collected through online research based on the classification of the German physician statistics used by the German Medical Association (outpatient: employed or self-employed, inpatient: leading position or non-supervisory). Geographical analysis of the local retention effect was carried out using a geographic information system.

**Results:**

Tracking reveals a significantly higher proportion of graduates working in outpatient health care (49.7%), which is due to the 69% increase in the rate of self-employment compared to the national average. The geographical analysis reveals a strong concentration of alumni in close proximity of the alma mater (retention effect), i.e. a 207-fold increase of the alumni density within a distance of 6 km around the UW/H. The local retention effect ranges approximately 30 km, where the increase is still 12-fold.

**Conclusions:**

The methodological approach presented in this study provides a novel way for tracking alumni and quantifying their spatial distribution patterns. By demonstrating how such data can be systematically analyzed, the study offers a practical framework for the evaluation of medical faculties and can help estimating the potential impact of e.g. newly established medical faculties on local healthcare provision. Hence, this study can serve as a blueprint for similar alumni tracking approaches.

## Zusammenfassung

**Hintergrund:**

Demografische und strukturelle Veränderungen im Gesundheitswesen haben in den letzten Jahren zu einem zunehmenden Ärzt:innenmangel in Deutschland geführt, insbesondere in ländlichen Gebieten. Medizinische Fakultäten nehmen zunehmend curriculare Anpassungen vor, um die Attraktivität dringend benötigter Stellen im Gesundheitswesen zu erhöhen. Das Tracking von Alumni ist für die Evaluierung von Lehrplänen und Optimierungsbemühungen und damit für die gezielte Verbesserung der Gesundheitsversorgung von entscheidender Bedeutung.

**Methoden:**

Daten zu den beruflichen Tätigkeiten von 333 Medizinabsolvent:innen der Universität Witten/Herdecke (UW/H) wurden mit einer Online-Recherche erhoben, die auf der Klassifizierung der deutschen Ärztestatistik basiert (ambulant: angestellt oder niedergelassen, stationär: leitende Position oder ohne Leitungsfunktion). Die geografische Analyse des Klebeeffektes wurde mithilfe eines geografischen Informationssystems durchgeführt.

**Ergebnisse:**

Das Tracking zeigt einen signifikant höheren Anteil von Alumni, die in der ambulanten Gesundheitsversorgung tätig sind (49,7%), was auf den im Vergleich zum nationalen Durchschnitt um 69% höheren Anteil an Niederlassungen zurückzuführen ist. Die geografische Analyse zeigt eine starke Häufung von Alumni in unmittelbarer Nähe ihrer Alma Mater (Klebeeffekt), d. h. eine 207-fache Erhöhung der Alumnidichte in einem Umkreis von 6 km um die UW/H. Der Klebeeffekt hat eine Reichweite von ca. 30 km und die Erhöhung ist in dieser Entfernung 12-fach.

**Schlussfolgerung:**

Die in dieser Studie vorgestellte Methodik bietet eine neuartige Möglichkeit, Alumni zu tracken und ihre räumliche Verteilung zu quantifizieren. Die hier präsentierte systematische Analyse bietet einen praktischen Rahmen für die Evaluierung von medizinischen Fakultäten und kann dazu beitragen, die potenziellen Auswirkungen, z. B. neu gegründeter medizinischer Fakultäten auf die lokale Gesundheitsversorgung, abzuschätzen. Somit kann diese Studie als Blueprint für ähnliche Ansätze zum Tracking von Alumni dienen.

## Introduction

Ensuring comprehensive access to medical care in rural areas is a considerable challenge, not only in Germany and considerable efforts have already been made to address this issue [1], [2], [3]. The main problem is the growing shortage of physicians, particularly in primary care and in rural regions. According to data from the National Association of Statutory Health Insurance Physicians (Kassenärztliche Bundesvereinigung – KBV), the number of solo primary care practices in Germany has declined by nearly 9,000 between 2010 and 2024, representing a decrease of 25.8% (data from the National Association of Statutory Health Insurance Physicians, as of 2024 [https://gesundheitsdaten.kbv.de/cms/html/17020.php]). Another trend is the change in the career preferences of young physicians [4]. Today, many prefer employment to self-employment, which makes it difficult to find successors for established medical practices. In addition, many employees opt for part-time models, which increases the demand for employment relationships. Only 57.5% of the 143,043 employed physicians were working full-time in 2024. This is 22% less than in 2014 (data from the National Association of Statutory Health Insurance Physicians, as of 2024 [https://gesundheitsdaten.kbv.de/cms/html/16400.php]).

In this context, graduate tracking is becoming increasingly important [5], [6], [7], [8]. By systematically recording and analyzing the professional careers of former students, valuable insights can be gained into their career paths and decisions. This information is essential for the evaluation of curricular incentives for certain fields of professional activity and hence for the targeted development of training programs. Overall, graduate tracking not only enables better planning and management of medical education, but also contributes to the development of long-term strategies for securing medical care, e.g. in rural areas.

Studies showed that growing up in a rural environment and getting a rural education are factors that encourage physicians to work in rural areas [9], [10], [11], [12], [13], [14]. Furthermore, the location of the medical school [15], [16] or the teaching/training hospital [17], [18] is a factor, as higher densities of physicians are found in the vicinity of educational institutions. This effect is called local retention effect or pipeline effect. Since previous studies on this topic refer to averaged data from entire districts or cities (data from municipalities, etc.) [15], [16], [17], [18], [19], [20], it was not possible to quantify the local retention effect and its fading at educational institutions in fine detail. This requires tracking at an individual level, as conducted in this study. Here we present a way to quantify the strength and reach of the local retention effect based on medical alumni of the University of Witten/Herdecke (UW/H). Although UW/H is located in the center of the Ruhr metropolitan region and the data obtained from UW/H alumni does not provide direct data on the strength of the local retention effect in rural areas, the methodology represents a valid approach to quantify this effect in detail.

The UW/H medical degree program has always been characterized by problem-based learning (PBL) and its strong practical focus as well as high patient-centeredness as well as the possibility for an integrated curriculum for anthroposophic medicine [21], [22]. Since increasing its capacity to 84 students per semester starting in the summer semester of 2019, UW/H has also been making a growing contribution in terms of numbers to address the emerging shortage of doctors. This study investigates the professional activities of graduates from 1999 to 2008. A comparison with the nationwide frequencies from the German physician statistics (Ärztestatistik der Bundesärztekammer) [https://www.bundesaerztekammer.de/baek/ueber-uns/aerztestatistik/2024] allows for a direct comparison of the data obtained. The geographical analysis of locations of professional activities carried out using free software and open geographical data demonstrates a methodological potential that can also provide an interesting basis for other faculties. The approach taken in this graduate tracking study can be seen as a guide for future studies, as such tracking will also become increasingly important in other areas and degree programs.

## Methods

### Online-research

To determine the career paths of medical school graduates from UW/H, an online search was conducted. This is common for such studies and usually yields the best return rates due to the often low response rates of online surveys [7], [8], [23], [24], [25], [26], [27]. The research was primarily conducted using search engines such as Google. Graduates’ names and combinations of the names and specific keywords (“Arzt”, “Praxis”, “Universität Witten/Herdecke”) were used for the online search. In addition, professional networks such as LinkedIn and XING were searched, as these platforms often contain up-to-date professional information [28]. Classifying the reliability of search results (reliable, unreliable, person not found) during the search process is a simple and efficient way to improve data quality. In search results classified as unreliable, a person with the respective name could be identified. However, a reliable attribution as a former student of UW/H was not possible. In reliable searches, the individuals were identified as UW/H graduates, e.g., on the basis of an online CV. Only data from graduates whose search results were classified as reliable were included in the analysis. In the present study, graduates from summer semester of 1999 to summer semester of 2008 were examined. This provides sufficient time after approbation for professional orientation and to finish postgraduate medical training to examine professional activity independently of the high initial professional turnover rates [29], [30].

### Comparison with German physician statistics

**Figure 1:**
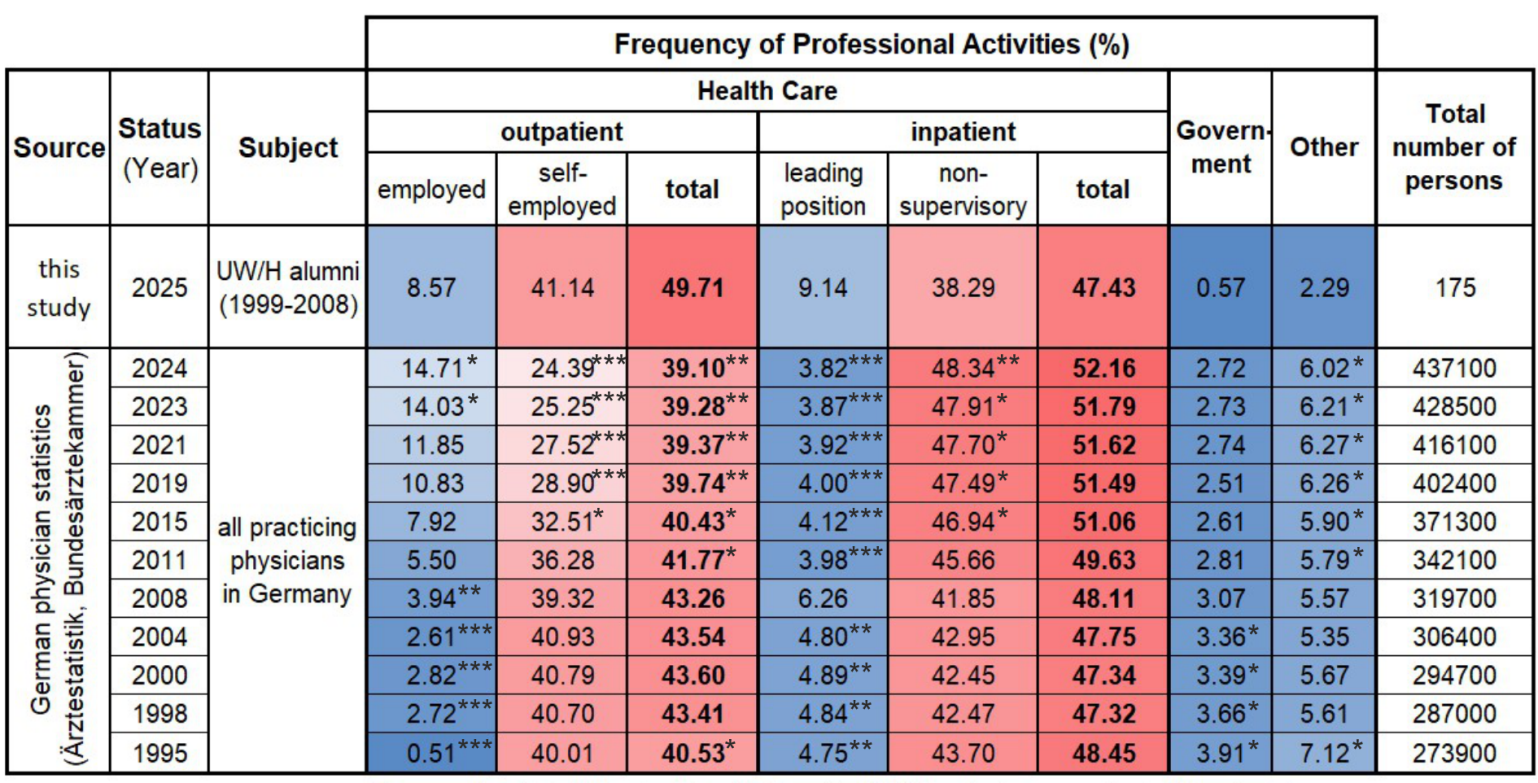
Heatmap showing the frequency of professional activities of UW/H alumni compared to the German physician statistics The heatmap color scale ranges from dark blue (minimum value) to dark red (maximum value). Frequencies that differ significantly from the UW/H cohort are marked with asterisks (Z-test for two proportions; *: p=0.05; **: p=0.01; ***: p=0.001).

Unlike many other studies, which perform postgraduate follow-ups in isolation for their own institution or a small circle of cooperating institutions [8], the data collection in this study was specifically adapted to the annual German physician statistics published by the German Medical Association, ensuring direct comparability with nationwide data. For professional activity, a comparison was made with the German physician statistic from December 2024, as this is closest to the time of the online research. Furthermore, results from earlier German physician statistics were included in figure 1 (Fig. 1) to visualize healthcare trends over the past 30 years. Similar to the German physician statistic, physicians working in inpatient care were categorized according to whether they held a leading position (leading position: directors and chief physicians) or not (*non-supervisory*). Physicians working in outpatient care were assigned to the categories of *self-employed* and *employed*. In addition, the categories of *government* and *others* were created for alumni not working in healthcare.

### Geographical analysis of professional activities

**Figure 2:**
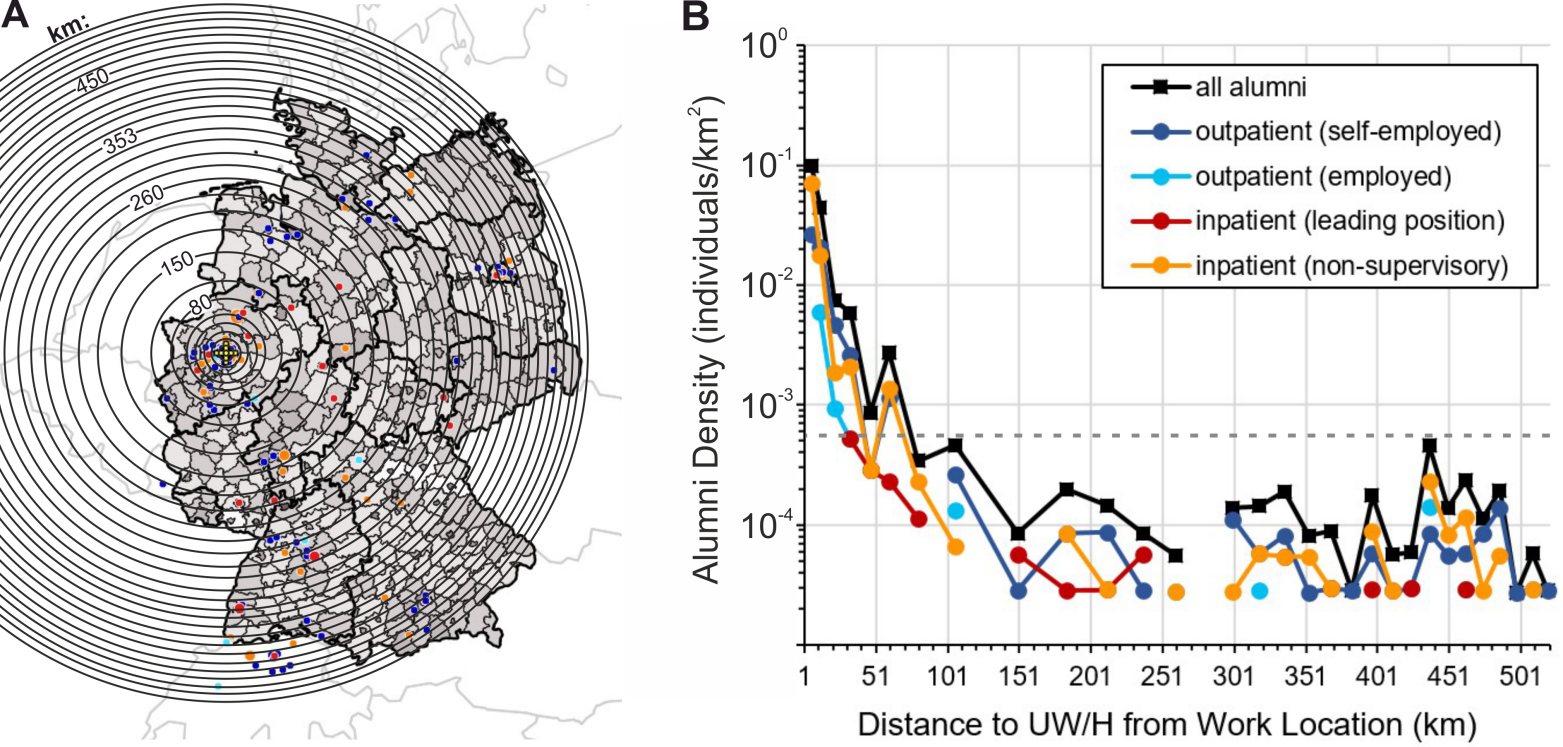
Alumni density and local retention effect A) Defined ring-shaped areas for the quantification of the alumni density in dependence of the distance to the UW/H. B) Determined alumni density. The gray dashed line indicates the hypothetical alumni density in case of homogeneous distribution across Germany.

The location of the practices or place of employment (address, postal code, city, and state) were collected in order to analyze the geographical distribution. Their geographical distribution was analyzed and visualized using a geographic information system (QGIS, version: 3.40.5-Bratislava). Open data from the OpenStreetMap project were used by the Federal Agency for Cartography and Geodesy (Data License Germany – Version 2.0) to display the state and district boundaries of Germany (federal states: [https://gdz.bkg.bund.de/index.php/default/open-data/verwaltungsgebiete-1-2-500-000-stand-31-12-vg2500-12-31.html]; administrative districts: [https://gdz.bkg.bund.de/index.php/default/digitale-geodaten/verwaltungsgebiete/verwaltungsgebiete-1-5-000-000-stand-01-01-vg5000-01-01.html]). For the color shading of districts based on physician density (number of physicians per 100,000 inhabitants), data from the National Association of Statutory Health Insurance Physicians [https://gesundheitsdaten.kbv.de/cms/html/16402.php] were used. The national borders of the countries neighboring Germany were obtained from the Geographical Information Systems for the COmmission of European Community (GISCO) via the Eurostat website [https://ec.europa.eu/eurostat/de/web/gisco/geodata/administrative-units/countries]. The addresses of the locations of professional activities were converted into geographical coordinates using Michael Minn’s Python plug-in MMQGIS [https://plugins.qgis.org/plugins/mmqgis/]. The linear distance (air-line distance) between these places and the UW/H were calculated using the distance matrix function in QGIS. The analysis of alumni density as a function of the distance between the workplace and the alma mater was carried out by determining the frequency of alumni in defined concentric circles around the UW/H. This is emphasized in figure 2 A (Fig. 2). The exact lengths of the radii of the circles enclosing the ring-shaped areas are: 6, 12, 22, 33, 47, 60, 80, 106, 150, 184, 212, 237, 260, 280, 300, 318, 336, 353, 368, 383, 397, 411, 424, 437, 450, 462, 474, 486, 498, 509, 520 km. The analysis was performed independently of the geographical coordinates based on the previously determined linear distance in Excel using automated counting with the COUNTIF function (e.g.: =COUNTIF(C$2:C$78;“<”&B96)-COUNTIF(C$2:C$78;“<”&B95); C$2:C$78: range with linear distances, B95: inner radius of the ring-shaped area, B96: outer radius of the ring-shaped area).

### Data analysis and visualization

Data collection, analysis, and visualization were performed using Excel (Microsoft Office Professional Plus 2016) and QGIS (version: 3.40.5-Bratislava) for geographical data. The statistical significance of two rates (compare figure 1 (Fig. 1)) was analyzed using the Z-test for two proportions and multiple group comparisons were made using the Kruskal-Wallis Test.

### Ethics vote

The Ethics Committee of Witten/Herdecke University approved this study (project number: S 114/2025).

## Results

### Professional activities

Of the 333 UW/H alumni, 176 (52.9%) could be reliably identified. Although women account for 59.5% of the 333 alumni, only 48.3% of the alumni identified through the research are female. The higher proportion of marriage-related name changes among women [31] is likely to be the main reason for this imbalance in the online traceability. Of the 176 individuals identified, 175 were working at the time of the research. The following results are based on these 175 professionally active alumni. Only 5 alumni (2.9%) are not working in healthcare, but employed in public authorities or as program coordinators in medical education. This is significantly less than the national average (2024 German physician statistic; p=0.0379). The remaining 170 alumni are divided almost equally between outpatient (87 individuals) and inpatient (83 individuals) healthcare (see figure 1 (Fig. 1)). Identified UW/H alumni are significantly less likely to pursue careers outside of healthcare than the national average (compared to the 2024 German physician statistic; p=0.0059). The frequency of physicians working in outpatient care among UW/H alumni is approximately 26% higher than the national average (2024 German physician statistic; p=0.004). This is due to the high proportion of physicians in private practice (41.1%), which is significantly higher than the national average at 24.4% (2024 German physician statistic; p<0.000000). There are no overall differences in inpatient care, although UW/H alumni are more likely to hold leading positions as directors or chief physicians (2024; German physician statistic; p=0.00024). Of these 16 alumni with leadership positions in inpatient healthcare, only two are female (12.5%). The proportion of women among employed alumni in outpatient healthcare is high with 80% (12 out of 15 people). In the other two categories, the gender ratio is balanced (proportion of women: inpatient (employed): 47.8%, outpatient (self-employed): 51.4%).

### Geographical distribution of locations of professional activity

**Figure 3:**
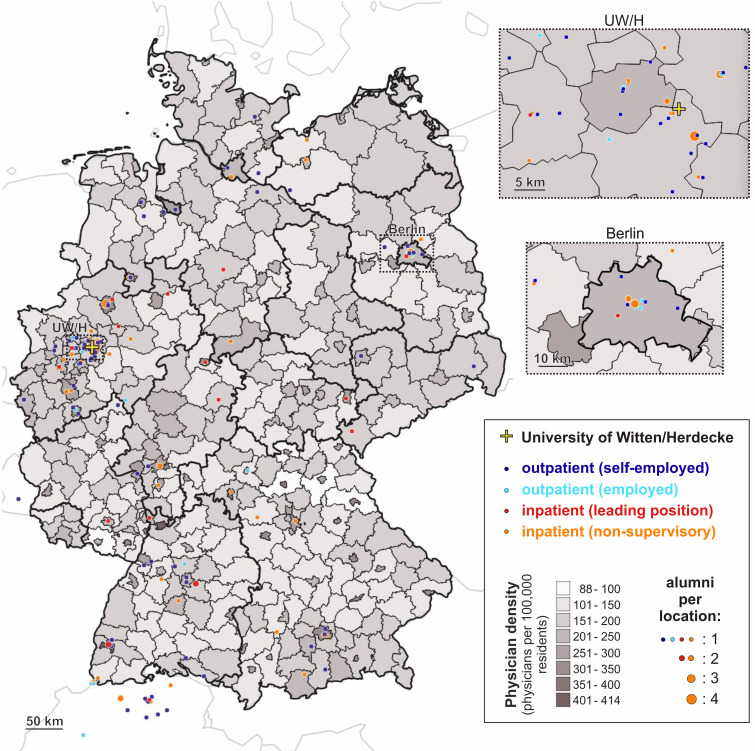
Geographical distribution of the locations of UW/H alumni's professional activities. The gray background indicates the general density of physicians in each district, and the size of the dots indicates the number of alumni at each location.

The geographical distribution of alumni working in healthcare was analyzed and visualized using a geographic information system (see figure 3 (Fig. 3)). A total of 168 alumni from mainland Europe were included, and their locations of professional activities (location of the clinic/practice) were marked according to their affiliation with one of the four groups from the German physician statistic. The alumni are spread across Germany, but there is a strong concentration in the densely populated Ruhr area, especially around UW/H. Further concentrations are in Berlin and the part of Switzerland bordering Germany around Zurich and Basel. A connection between alumni working in Berlin and Basel and the anthroposophical clinics there (GKH Havelhöhe and Klinik Arlesheim) was checked due to the possibility of an additional qualification in anthroposophic medicine at the UW/H, but could not be found. Apart from some practices in rural districts such as Altenkirchen, Havelland, Sigmaringen, or Herzogtum Lauenburg, the distribution patterns of UW/H alumni resemble the nationwide distribution trends mentioned at the beginning, with increased numbers working in metropolitan regions or close to metropolitan areas.

### Quantification of the local retention effect 

Since alumni tend to work close to their alma mater [15], [18] and there is no precise quantification of the extent of this effect to date, we determined the alumni density as a function of distance from UW/H. In the case of a homogeneous distribution of the 168 alumni throughout Germany, the alumni density would be 0.00047 alumni/km^2^ (dashed gray line in figure 2 B (Fig. 2)). The UW/H alumni densities determined for the ring-shaped areas are above this theoretical value within a radius of 60 km (r_0_ to r_60_=0.00531 alumni/km^2^). Within this 60 km radius, the alumni density decreases 35-fold from the innermost circle with a radius of 6 km (r_0_ to r_6_=0.09726 alumni/km^2^) to the ring with a radius of 47 to 60 km (r_47_ to r_60_=0.00275 alumni/km^2^). The density outside this 60 km radius is 3.6 times lower than that of the theoretical uniform distribution (r_60_ to r_520_=0.00013 alumni/km^2^). This shows a strong concentration within a very small radius of 12 km around the UW/H (factor of density increase compared to the theoretical uniform distribution: r_0_ to r_6_: 207; r_6_ to r_12_: 94; r_12_ to r_22_: 16; r_22_ to r_33_: 12; r_33_ to r_47_: 1.8; r_47_ to r_60_: 5.8). The decrease in the concentration factor with increasing distance from UW/H appears similar in all four groups of professional activities. The mean distances from UW/H to the location of professional activity are 222±184 km (outpatient (self-employed)), 261±192 km (outpatient (employed)), 223±139 km (inpatient (leading position)), and 220±189 km (inpatient (non-supervisory)) and the four groups doesn’t differ significantly (Kruskal-Wallis Test, p=0.76521).

## Discussion

Tracking former students is important for evaluating degree programs and changes to the curriculum [6], [7], [28], [32], [33], [34], but also for determining the success of individual projects and courses, such as rural doctor programs or summer schools [5], [35]. By taking additional parameters into account, such as examination results or duration of study, further exciting insights can be gained [36].

Tracking graduates is associated with various challenges. For example, the availability of alumni decreases over time as contact details change and are seldom updated. Alumni email addresses are also used or accessed less frequently, which makes reliable communication difficult [37]. It is therefore not surprising that the response rate for data collection via surveys is rather low [10], [12], [26], [27]. The alternative is time-consuming and labor-intensive research, which, thanks to increasing digitalization and professional social media presence (e.g., LinkedIn or XING), enables higher hit rates of mostly over 60% [7], [24], [25]. Without constant and time-consuming maintenance of alumni databases, it will be difficult to achieve hit rates close to 100%, e.g., due to name changes following marriage. Even if the research is mainly carried out by temporary staff with low hourly wages (as in the present study), high costs can still arise. For example, 50,000 Canadian dollars were spent on research work carried out by temporary staff for the alumni tracking of the University of Toronto’s 10,000 PhD project [25]. Another problem with tracking graduates is the lack of comparability between individual studies [8]. Orientation on nationwide records, such as the German physician statistics, is a good way to improve the comparability of different studies. A uniform, centralized, nationwide approach (e.g., by including the universities and places of professional activities in the German physician statistic, followed by an automated analysis at the level of the individual faculties) could provide all medical faculties with important feedback on the outcomes of their faculties in a more efficient manner.

Alumni tracking studies usually focus on analyzing professional activities. An analysis of geographical location is less common and is usually based on officially available averaged data from authorities or district administrations [15], [16], [17], [18], [19], [20]. The analysis of alumni distribution at the level of individual locations of professional activities examined here was able to demonstrate for the first time the strong local retention effect in vicinity of the educational institution. The distribution of alumni and the retention effect also depends on regional factors. If, for example, a medical school is located in a rural town, the potential lack of clinics outside the town will inevitably lead to a drastic reduction in the density of stationary employed alumni. In contrast, UW/H is located in the densely populated Ruhr area with a high demand for medical professionals. Since UWH alumni are more prevalent in densely populated regions with an academic environment (e.g., the Ruhr area, Berlin, Zurich/Basel), the general preference of medical students for metropolitan areas [1], [2], [3] can be reconfirmed. The observed concentration of alumni around the UW/H is therefore based on the region's sociocultural characteristics. The fact that the alumni density decreases more than tenfold within a radius of about 20 km, despite the metropolitan character of the included area and the presence of up to approximately 60 clinics as potential employers, for example, suggests that the local retention effect additionally contributes to the observed concentration of alumni in close proximity of the UW/H. In order to assess the scope of the retention effect more accurately, further studies of this kind are needed in other regions with different local conditions. Faculties in rural areas are particularly interesting in this regard. This allows for a better assessment of the influence of different local conditions on the retention effect and thus the impact of the location of a medical school on the local physician density. A medical school in a rural area could offer benefits to the region not just because of the retention effect. Since students would live in a rural area for several years during their studies, and this is a key predictor of future employment in rural areas [9], [10], [11], [12], [13], [14], this could have a synergistic effect together with the retention effect. Strong integration of surrounding clinics and practices into training represents a further opportunity to strengthen local healthcare provision in the long term.

The analysis of the professional activities of the 175 alumni shows an above-average employment rate in outpatient care, especially in private practices. In inpatient care, leading positions among alumni are more than twice as frequent as the current national average (German physician statistic, 2024). This could be related to competence-oriented teaching [21], as communication and teamwork skills are crucial leadership competencies [38]. The possibility of a discovery bias should also be considered when interpreting the frequencies of professional activities presented here, as individual groups, such as directors and chief physicians, may have higher online discoverability and thus lead to an overestimation. The low proportion of women in leadership positions is consistent with the prevailing gender gap in the clinical setting [39], [40].

We would like to point out that the results of this study are hard to generalize. The UWH's model program is different from the curriculum at national universities. PBL is the leading approach for instruction and clinical training takes place in a decentralized manner in numerous cooperating clinics spread across a wide geographical area rather than in a central university hospital [21], [22]. In addition, students are not selected on the basis of their grades, but undergo a special selection process involving several interviews, in which their attitude and motivation are assessed in addition to their academic aptitude [41]. The differences in the selection and training of students could have a significant impact on their career paths. The location of UW/H in the center of a large metropolitan area must also be taken into account for the interpretation of the observed alumni distribution. Therefore, additional faculties are encouraged to conduct comparable studies to improve our understanding of the post-graduation pathways and regional distribution of alumni. If larger numbers of alumni can be gathered, for example in larger faculties, it might also be statistically feasible to analyze individual medical specialties. Moreover, further studies could analyze the alumni's motivations for their respective career decisions (activity and choice of location) to improve our understanding of student’s professional decision making. 

## Conclusions

Two key aspects characterize the approach presented here for tracking former medical students. On the one hand, the combination with the nationwide German physician statistics enables the classification of the data obtained. On the other hand, the geographical tracking of alumni's workplaces at an individual level allows the quantification of the strength and reach of the local retention effect for the first time and enables the estimation of the meaning of a medical school's location. The retention effect should also be quantified in rural regions, which will improve understanding of the retention effect under different regional circumstances. These findings are of great importance for the evaluation of medical faculties or, for example, for the selection of the locations of new medical schools or teaching hospitals to improve local medical care.

## Acknowledgements

The authors would like to thank Anke Bauske (Head of Student Affairs, UW/H) for providing the lists of graduates' names.

## Notes

### Author contributions

R.H conceived and designed research; S.M., S.C.R., and R.H. performed online research; R.H. analyzed data; R.H., M.H., and A.H. interpreted research results; R.H. prepared figures and drafted manuscript; R.H., A.H. and M.H. edited and revised manuscript; R.H., S.M., S.C.R., A.H., and M.H. approved final version of manuscript.

### Authors’ ORCIDs

- Robin Herbrechter: [0000-0002-2857-9136]
- Stefanie Mattern: [0009-0008-0274-8262]
- Sophie-Charlotte Rosenberger: [0009-0000-0367-6506]
- Annika Haupt: [0009-0000-6090-3576]

## Competing interests

The authors declare that they have no competing interests. 

## References

[1] Rosenthal MB, Zaslavsky A, Newhouse JP (2005). The geographic distribution of physicians revisited. Health Serv Res.

[2] Elma A, Nasser M, Yang L, Chang I, Bakker D, Grierson L (2022). Medical education interventions influencing physician distribution into underserved communities: a scoping review. Hum Resour Health.

[3] Longenecker RL, Andrilla CHA, Jopson AD, Evans DV, Schmitz D, Larson EH, Patterson DG (2021). Pipelines to Pathways: Medical School Commitment to Producing a Rural Workforce. J Rural Health.

[4] Gigou S, Corazza L, Fett S, Tauscher M, Gerlach R, Donnachie E, Schneider A (2024). Entwicklung der Ärzt*innenzahlen und Beschäftigungsverhältnisse in Bayern – Trends in den Ärzt*innenstatistiken der Bayerischen Landesärztekammer und der Kassenärztlichen Vereinigung Bayerns. Gesundheitswesen.

[5] Desmond RA, Padilla LA, Daniel CL, Prickett CT, Venkatesh R, Brooks CM, Waterbor JW (2016). Career Outcomes of Graduates of R25E Short-Term Cancer Research Training Programs. J Cancer Educ.

[6] Akabas MH, Brass LF (2024). The National MD-PhD Program Outcomes Study: career paths followed by Black and Hispanic graduates. JCI Insight.

[7] Lee CC, Vear A, Howard B, Choate J (2025). Tracking graduate outcomes of undergraduate physiology major students. Adv Physiol Educ.

[8] Krasna H, Gershuni O, Sherrer K, Czabanowska K (2021). Postgraduate Employment Outcomes of Undergraduate and Graduate Public Health Students : A Scoping Review. Public Health Rep.

[9] Farmer J, Kenny A, McKinstry C, Huysmans RD (2015). A scoping review of the association between rural medical education and rural practice location. Hum Resour Health.

[10] Kondalsamy-Chennakesavan S, Eley DS, Ranmuthugala G, Chater AB, Toombs MR, Darshan D, Nicholson GC (2015). Determinants of rural practice: positive interaction between rural background and rural undergraduate training. Med J Aust.

[11] Pagaiya N, Kongkam L, Sriratana S (2015). Rural retention of doctors graduating from the rural medical education project to increase rural doctors in Thailand: a cohort study. Hum Resour Health.

[12] Kwan MM, Kondalsamy-Chennakesavan S, Ranmuthugala G, Toombs MR, Nicholson GC (2017). The rural pipeline to longer-term rural practice: General practitioners and specialists. PLoS One.

[13] O'Sullivan BG, McGrail MR, Russell D, Chambers H, Major L (2018). A review of characteristics and outcomes of Australia's undergraduate medical education rural immersion programs. Hum Resour Health.

[14] Magnus JH, Tollan A (1993). Rural doctor recruitment: does medical education in rural districts recruit doctors to rural areas?. Med Educ.

[15] Brokaw JJ, Mandzuk CA, Wade ME, Deal DW, Johnson MT, White GW, Wilson JS, Zollinger TW (2009). The influence of regional basic science campuses on medical students' choice of specialty and practice location: a historical cohort study. BMC Med Educ.

[16] Park K, Kim H, Lee J, Shin J, Park A (2023). Determinants of Working Practice Location for Clinicians According to High School, Medical School, and Resident Training Locations in Korea. Healthcare (Basel).

[17] McGrail MR, O'Sullivan BG (2021). Increasing doctors working in specific rural regions through selection from and training in the same region: national evidence from Australia. Hum Resour Health.

[18] Russo G, Ferrinho P, de Sousa B, Conceição C (2012). What influences national and foreign physicians' geographic distribution? An analysis of medical doctors' residence location in Portugal. Hum Resour Health.

[19] Scholz S, Graf von der Schulenburg JM, Greiner W (2015). Regional differences of outpatient physician supply as a theoretical economic and empirical generalized linear model. Hum Resour Health.

[20] Bauer J, Brueggmann D, Ohlendorf D, Groneberg DA (2016). General practitioners in German metropolitan areas - distribution patterns and their relationship with area level measures of the socioeconomic status. BMC Health Serv Res.

[21] Schlett CL, Dahmen HD, Polacsek O, Federkeil G, Fischer MR, Bamberg F, Butzlaff M (2010). Job requirements compared to medical school education: differences between graduates from problem-based learning and conventional curricula. BMC Med Educ.

[22] Frost K, Edelhäuser F, Hofmann M, Tauschel D, Lutz G (2019). History and development of medical studies at the University of Witten/Herdecke - an example of "continuous reform". GMS J Med Educ.

[23] Daniel CL, Michael Brooks C, Waterbor JW (2011). Approaches for longitudinally tracking graduates of NCI-funded short-term cancer research training programs. J Cancer Educ.

[24] McMahon M, Habib B, Tamblyn R (2019). The Career Outcomes of Health Services and Policy Research Doctoral Graduates. Healthc Policy.

[25] Reithmeier R, O'Leary L, Zhu X, Dales C, Abdulkarim A, Aquil A, Brouillard L, Chang S, Miller S, Shi W, Vu N, Zou C (2019). The 10,000 PhDs project at the University of Toronto: Using employment outcome data to inform graduate education. PLoS One.

[26] Leider JP, Rockwood TH, Mastrud H, Beebe TJ (2024). Engaging Public Health Alumni in the Tracking of Career Trends: Results From a Large-Scale Experiment on Survey Fielding Mode. Public Health Rep.

[27] Zuniga NC, Colbern A (2021). The Annual BUILD Snapshot: Tracking Alumni Outcomes. HCI Intern 2021 Late Break Pap (2021).

[28] Case TL, Gardiner A, Rutner P, Dyer JN (2013). A LinkedIn Analysis of Career Paths of Information Systems Alumni. J South Assr Inform Syst.

[29] Chen AS, Leet JG, Schneider B, Teramoto M, Abdullah NM, McCormick ZL (2024). Physician turnover rates and job stability in interventional spine and pain practices: Results of an IPSIS survey study. Interv Pain Med.

[30] Misra-Hebert AD, Kay R, Stoller JK (2004). A review of physician turnover: rates, causes, and consequences. Am J Med Qual.

[31] Shafer EF, Christensen MA (2018). Flipping the (Surname) Script: Men’s Nontraditional Surname Choice at Marriage. J Fam Issues.

[32] Silva EA, Mejia AB, Watkins ES (2019). Where Do Our Graduates Go? A Tool Kit for Tracking Career Outcomes of Biomedical PhD Students and Postdoctoral Scholars. CBE Life Sci Educ.

[33] Glynn LG, Regan AO, Casey M, Hayes P, O'Callaghan M, O'Dwyer P, Culhane A, Cuddihy J, Connell BO, Stack G, O'Flynn G, O'Donnell P, O'Conner R, McKeague H, Mc Grath D (2021). Career destinations of graduates from a medical school with an 18-week longitudinal integrated clerkship in general practice: a survey of alumni 6 to 8 years after graduation. Ir J Med Sci.

[34] Reilly JM, Edge I, Greenberg I (2024). Where Are They Now? Alumni Outcomes From a Medical School Primary Care Pathway Program. Fam Med.

[35] Jamar E, Newbury J, Mills D (2014). Early career location of University of Adelaide rural cohort medical students. Rural Remote Health.

[36] Lightfoot RC, Doerner WG (2008). Student Success and Failure in a Graduate Criminology/Criminal Justice Program. Am J Crim Justice.

[37] Padilla LA, Venkatesh R, Daniel CL, Desmond RA, Brooks CM, Waterbor JW (2016). An Evaluation Methodology for Longitudinal Studies of Short-Term Cancer Research Training Programs. J Cancer Educ.

[38] Bornman J, Louw B (2023). Leadership Development Strategies in Interprofessional Healthcare Collaboration: A Rapid Review. J Healthc Leadersh.

[39] Saadoun R, Risse E, Sadoun L, Kamal A, Pudszuhn A, Obermueller T (2023). Gender distribution and women leadership in German Otolaryngology, Head and Neck Surgery. Laryngoscope Investig Otolaryngol.

[40] Weiss M, Dogan R, Eisenberg U, Velalakan A, Krüger J, Moritz I, Nistor-Gallo D, Flueh C, Janz C, Ahmadi R, Hakvoort K, Forster MT, “Women in Neurosurgery - Open for all” - Official Commission of the German Society of Neurosurgery (DGNC) (2023). Path to success: female leaders in German neurosurgery. Neurosurg Rev.

[41] Bokelmann A, Ehlers JP, Zupanic M (2023). Multimodal selection of medical students: The predictive power of individual process components in the two-stage selection process at Witten/Herdecke University (UW/H). Z Evid Fortbild Qual Gesundhwes.

